# Aerogel-Lined Capillaries for Raman Signal Gain of Aqueous Mixtures

**DOI:** 10.3390/s22124388

**Published:** 2022-06-10

**Authors:** Felix Spiske, Martin Peter Dirauf, Andreas Siegfried Braeuer

**Affiliations:** Institute of Thermal, Environmental and Resources’ Process Engineering (ITUN), Technische Universität Bergakademie Freiberg, 09599 Freiberg, Germany; felix.spiske@tun.tu-freiberg.de (F.S.); martin.dirauf@tun.tu-freiberg.de (M.P.D.)

**Keywords:** Raman spectroscopy, optofluidic waveguide, Raman sensor, aerogel, liquid core waveguide, backward scattering, waveguide capillary cell, metal-lined hollow-core fibre

## Abstract

We report an experimental study on the gain of the Raman signal of aqueous mixtures and liquid water when confined in aerogel-lined capillaries of various lengths of up to 20 cm and various internal diameters between 530 and 1000 µm. The lining was made of hydrophobised silica aerogel, and the carrier capillary body consisted of fused silica or borosilicate glass. Compared to the Raman signal detected from bulk liquid water with the same Raman probe, a Raman signal 27 times as large was detected when the liquid water was confined in a 20 cm-long capillary with an internal diameter of 700 µm. In comparison with silver-lined capillaries of the same length and same internal diameter, the aerogel-lined capillaries featured a superior Raman signal gain and a longer gain stability when exposed to mixtures of water, sugar, ethanol and acetic acid.

## 1. Introduction

Linear Raman spectroscopy is a species-specific, quantitative, experimentally simple and non-invasive spectroscopic method that does not require any sample preparation, and thus qualifies for online process analysis [1,2,3,4]. Unfortunately, due to the inherently small Raman scattering cross section, Raman signal yield is low [5], which can be encountered by several measures. The most obvious measure is increasing the excitation laser power, but the power of available lasers might be limited, and excitation powers will modify or destroy the matter in the probe volume if they are too large. In chemical process plants, the environment of the measurement location often limits the allowed laser powers, such as in potentially explosive atmospheres (ATEX). Therefore, various other measures are known to increase the Raman signal, which relies on non-linear light-matter interactions [6], surface-enhanced strategies [7], multiple excitation strategies [8] or the confinement of light and analyte in channels [9]. Non-linear excitation strategies are not further regarded here because of two reasons: Regarding linear Raman spectroscopy, more elaborate experimental setups are required, and signal quantification is more complex. Surface-enhanced strategies are also not further regarded here as, again, signal quantification is more complex, and plasmonic particles have to be introduced into the measurement volume. In multi-pass cells, excitation laser radiation is launched multiple times through the same probe volume. The multi-pass arrangement is realised by aligning concave mirrors along the circumference of a circle. Once the laser beam is launched into the multi-pass cell, it is reflected multiple times through the probe volume (the centre region of the circle) before it leaves the multi-pass cell again. Consequently, the matter in the probe volume is effectively excited multiple times. Configurations such as this have also proven to be applicable outside of research labs in field experiments or in industrial environments for liquid and gaseous matter [10,11,12,13,14,15,16,17]. Alternative confinement strategies utilise channels, which simultaneously serve as fluid and light guides. Consequently, the Raman signal is generated all the way through the channel and is guided along the channel, exiting through one of its two ends. All the fluid volume inside the channel can be considered as probed volume. Therefore, these strategies are understood as methods which enlarge the probed volume. Fluid- and light-guiding channels are realised as hollow-core photonic crystal fibres, as gold- or silver-lined capillaries or, when the fluid is in the liquid aggregation state, as liquid-core step-index waveguides. Hollow-core photonic crystal fibres possess hollow-core diameters typically from 5 to 30 µm [18,19,20], and thus are prone to clogging, especially when liquid analytes of an industrial grade are guided through them. Gold- or silver-lined capillaries are not prone to clogging, as they are available with larger inner diameters. However, inorganic solutes can deposit from the liquid on the highly reflective linings (scaling) and cause a gradual worsening of the reflection properties. Next to scaling, micro-organisms growing on the lining (fouling) also reduce the reflection properties of the lining. In liquid-core step-index waveguides, the liquid analyte is the core, which is surrounded by a cladding of a lower index of refraction. A liquid stream surrounded by air is an example of a liquid-core step-index waveguide [21]. 

Several groups report optofluidic step-index waveguides, which were based on hydrophobised silica aerogel [22,23,24,25,26,27,28]. Silica aerogel is a material which features indices of refraction close to those of air. While Erkey et al. [23] produced these optofluidic waveguides by manually drilling channels into aerogel blocks, Fraval et al. [22] produced them by coating the inner surface of capillaries with silica aerogel linings. Advanced applications of these aerogel-based optofluidic waveguides have been reported, where the light induces a reaction in the aqueous mixture (photochemistry) [25], or where chemical compounds in the aqueous mixture are detected by analysing the spectrum of the transmitted light (absorption spectroscopy) [24,25].

Inspired by what has already been reported, we report an experimental study that shows the suitability of fused silica capillaries lined with hydrophobic silica aerogel for gaining a Raman signal from pure water and aqueous solutions of glucose, ethanol and acetic acid. Furthermore, we draw an experimental comparison with silver-lined capillaries: firstly, with regard to the Raman signal gain in pure water at equal capillary dimensions, and secondly, considering the gain stability when exposed to aqueous solutions of glucose, ethanol and acetic acid, also at equal capillary dimensions.

## 2. Materials and Methods

### 2.1. Materials

The solvents methanol, ethanol and n-hexane, as well as the concentrated ammonium water (25 wt%) and the silica gel precursor, tetramethylorthosilicate (TMOS), were purchased from Sigma-Aldrich/MERCK. Acetic acid was also obtained from Sigma-Aldrich/MERCK. Glucose was procured from a local grocery. The silver-lined capillaries were purchased from DOKO Engineering Japan. They are 20 cm long and have inner diameters of 700 µm. The fused silica capillaries with circular cross sections were obtained from MOLEX and have inner/outer diameters of 530 ± 10 µm/700 ± 20 µm and 700 ± 10 µm/850 ± 20 µm, respectively. The borosilicate glass capillary with an internal diameter of 1000 µm possesses a steel body and has a circular cross section. It was purchased from Chemietechnik Reichelt. Hexamethyldisilazane (HMDS) was provided by Sigma-Aldrich/MERCK and utilised as a hydrophobising agent.

### 2.2. Generation of the Gel–Particle Dispersion

The gel–particle dispersion is required for generating the aerogel lining on the inner surface of the fused silica capillaries. It is composed of the solvent ethanol and the dispersed silica alcogel particles and was produced based on the recipe described by Fraval et al. [22]. Therefore, 25 wt% concentrated ammonium water was thinned to obtain a 0.025 M aqueous solution of NH_4_OH. Afterwards, 5 mL of the silica gel precursor TMOS, 40 mL methanol and 2.5 mL of the 0.025 M ammonium water were presented in a sealable glass beaker. The beaker was placed into an oven at 50 °C for 48 h to induce the aging of the silica gel. Subsequently, the aged gel was crushed using a spatula and overlaid with 50 mL methanol, which was immediately followed by placing the beaker back into the oven at 50 °C for 1 h. Afterwards, the methanol was decanted and replaced by 50 mL of fresh methanol, followed by placing the beaker at 50 °C for 1 h in the oven. After decanting the methanol a second time, the washing procedure was repeated twice, overlaying the crushed gel with 50 mL of n-hexane instead of methanol. The n-hexane layer was decanted after the second washing step and replaced by a pre-mixed solution of 47.5 mL n-hexane and 2.5 mL HMDS (hydrophobising solution). The crushed gel, overlaid with the hydrophobising solution, was then placed into the oven at 50 °C for 24 h. Subsequently, the hydrophobising solution was decanted, followed by a repeat of the two-step washing procedure with n-hexane, as described above. Finally, the two-step washing procedure was analogously repeated using ethanol, where the ethanol layer was decanted. The residuum was then overlaid with ethanol at a ratio of 1 to 1 by mass. Finally, to obtain the gel–particle dispersion, which can be used for lining the capillaries, the ethanol-overlaid residuum was re-liquefied for 40 min, utilising a Fisherbrand FB120 ultrasonic homogeniser (120 W, 60% amplitude, 3 s pulse).

### 2.3. Fabrication of Aerogel-Lined Capillaries

**Figure 1:**
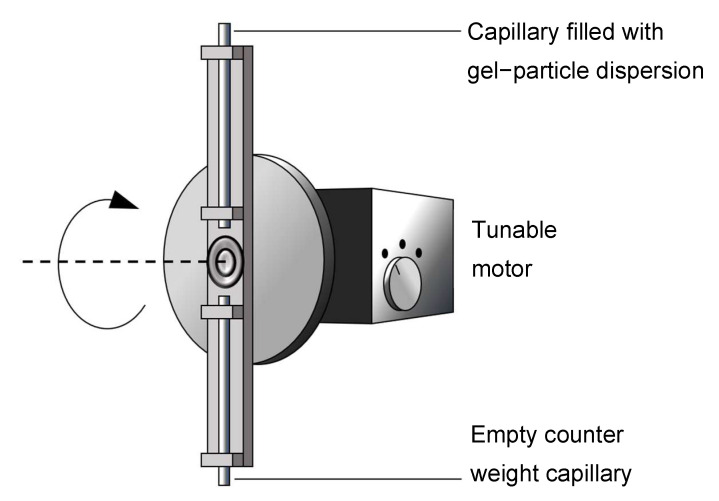
Sketch of the spin coater.

The aerogel lining was coated on the inner surface of the fused silica capillaries using a self-engineered spin coater, as sketched in Figure 1. The fused silica capillaries were not pretreated before lining with aerogel. They were first mounted into the spin coater and then filled with the gel–particle dispersion. Afterwards, the spin coater rotated the capillaries: first at a rotation speed of 240 rounds per minute for 10 s, then at 2000 rounds per minute for 60 s. The slow rotation drove the dispersion out of the capillaries and left behind a layer of the gel–particle dispersion wetting the inner surface of the capillary. The fast rotation drove air through the capillary, which dried the ethanol from the dispersion film and the alcogel particles. As a result, the inner surface of the capillary became lined with a dry layer of hydrophobised aerogel particles.

### 2.4. Experimental Setup for Raman Measurement of Aqueous Mixtures

**Figure 2:**
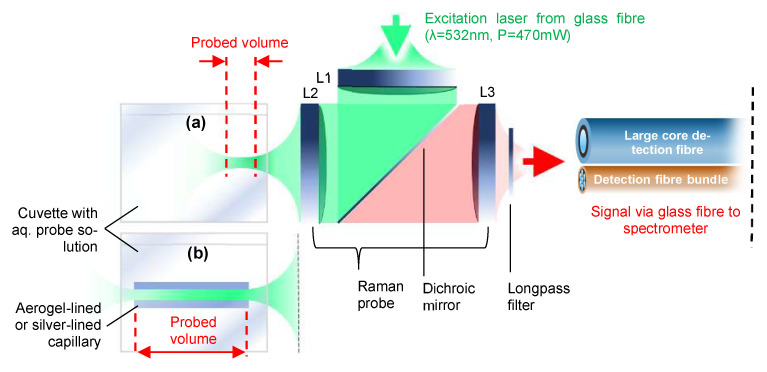
Sketch of the measurement setup with Raman probe, cuvettes with aqueous probe solutions and detection fibres (**b**) with and (**a**) without aerogel or silver-lined capillary.

Figure 2 illustrates the experimental setup which was used for measuring the Raman spectra of bulk aqueous analytes (Figure 2a) or, alternatively, an aqueous analyte confined inside the aerogel-lined capillary (Figure 2b). For the comparison of the aerogel-lined capillary with the silver-lined capillary, the aerogel-lined capillary was replaced with the silver-lined capillary. The following descriptions are provided for the aerogel-lined capillary and can be transferred to the silver-lined capillary on a one-to-one basis: The laser light (Cobolt Samba 1000, λ = 532 nm, P = 23.2 mW) is coupled into the Raman probe from the top via a silica fibre with a core diameter of ∼200 µm. The laser radiation is then collimated by lens L1 (f_1_ = 200 mm, ∅_1_ = 50 mm), reflected by the dichroic mirror and then focused into the aqueous solution (or liquid water) by lens L2 (f_2_ = 100 mm, ∅_2_ = 50 mm). Lenses L1 and L2 image the exit plane of the laser fibre with a magnification of M_L1,L2_ = −0.5 onto the entrance plane of the aerogel-lined capillary. Therefore, the diameter of the focused laser radiation in the entrance plane of the aerogel-lined capillary is ∼100 µm. The signals emerging from the probed volume are collected in a backscattering configuration through lens L2. The red-shifted Raman Stokes signals are purified by elastically scattered laser-light: firstly by the dichroic mirror, and secondly by the long pass filter (Raman razor edge RU 532 from Semrock). The transmitted Raman signals are coupled into a detection fibre bundle via lens L3 (f_3_ = 100 mm, ∅_3_ = 50 mm). The fibre bundle contains seven glass fibres with core diameters of 100 µm. They are aligned in a round-to-linear configuration, with the round end facing the Raman probe and the linear end matching the entrance slit of the dispersive spectrometer (Ocean Optics QEPro). At the round end, the light-accepting area of the detection fibre bundle features a diameter of ∼355 µm. Lenses L2 and L3 image the entrance plane of the aerogel-lined capillary with a magnification of M_L2,L3_ = −1 onto the round end of the detection fibre bundle. In order to find out whether the internal diameter of the aerogel-lined capillary affects the Raman signal gain or not, the round-to-linear fibre bundle was replaced with a large core diameter (1000 µm) glass fibre.

## 3. Results

### 3.1. Fabrication of Aerogel-Lined Capillaries

We observed that aerogel-lined capillaries, which were lined only once, did not provide reproducible light-guiding properties. Aerogel-lined capillaries with reproducible light-guiding properties were obtained when the lining procedure was repeated N = 5 times or more often. By visual observation, the aerogel-lined capillaries cannot be differentiated from the fused silica capillaries before the lining. 

### 3.2. Analysis of Raman Signal Gain of Aerogel-Lined and Silver-Lined Capillaries in Liquid Water

**Figure 3:**
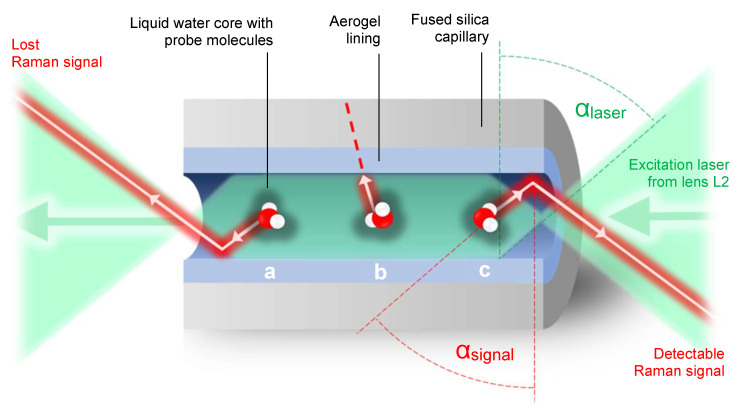
Sketch of the paths of the laser radiation (green) and potential red-shifted Raman signal rays through the aerogel-lined capillary.

Figure 3 illustrates the path of the laser radiation and the paths of the Raman signal radiation inside the aerogel-lined capillaries. The core of the waveguide is the liquid aqueous mixture, which features an index of refraction similar to that of water n_water_ ≈ 1.331 [29] (@ 650 nm, 298.15 K, 0.1 MPa). The cladding of the waveguide is the hydrophobised and highly porous silica aerogel, which features indices of refraction between the index of refraction of air and silica. The porosity of silica aerogels can be up to 99.99% [30], meaning that 99.99% of the volume of the aerogel is air, and the rest is silica. For highly porous aerogels, we consider the index of refraction of n_aerogel_ ≈ 1.0026 [31,32] (@ 632.8 nm, ambient conditions). According to Snell’s law and considering the above-mentioned indices of refraction n_water_ and n_aerogel_, the total internal reflection (TIR) on the interface between the liquid aqueous core and the surrounding aerogel is met for angles of incidence larger than the critical angle α_crit_ = 48.9°. This configuration serves as both a light and liquid guide. The hydrophobicity of the silica aerogel lining promises to prevent both the fouling and penetration of the liquid analyte into the air-filled pores of the aerogel.

Figure 3 shows that focused green laser radiation is incident to the inner surface of the capillary with angles of incidence larger than α_laser_ = 71.3°. The value of α_laser_ was computed from the focal length of lens L2, the diameter of the excitation laser beam (∼50 mm) before it passes through lens L2 and the indices of refraction of water (inside the cuvette) and air (outside the cuvette). As α_laser_ > α_crit_, TIR assures the guidance of the laser radiation along the capillary. The reference also shows potential paths of red-shifted Raman signal rays inside the aerogel-lined capillary. In case “a”, the Raman signal undergoes TIR, but leaves the capillary on the side averted to the Raman probe. In case “b”, the Raman signal is incident to the aerogel lining at an angle smaller than the critical angle, and thus does not undergo TIR and is extinguished. In case “c”, the Raman signal undergoes TIR and exits the capillary through the side facing the Raman probe. If it exits this side of the capillary with angles of incidence α_signal_ ≥ α_laser_, it is collected by lens L2 and can be detected.

**Figure 4:**
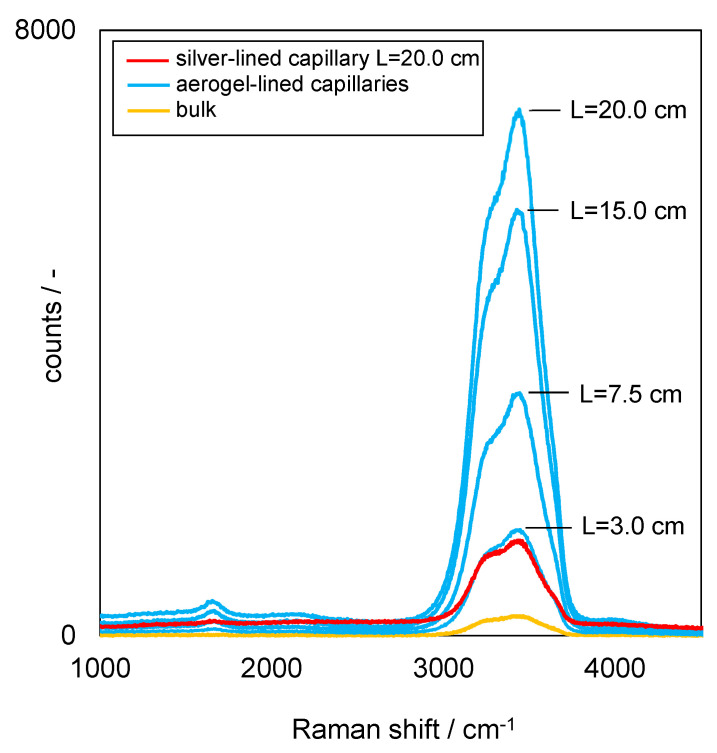
Raman spectra detected from pure bulk liquid water and utilising aerogel- or silver-lined capillaries for Raman signal gain.

Figure 4 shows the resulting Raman spectra from aerogel-lined capillaries of various lengths, from the silver-lined capillary and from the pure bulk liquid water not confined in any capillary (Figure 2a). The measurements are the results of a Raman signal integration time of 200 ms per spectrum. Both the aerogel-lined capillaries and the silver-lined capillary feature an internal diameter of ∅ = 700 µm.

As can be deduced from Figure 4, both the aerogel-lined capillaries (blue) and the silver-lined capillary (red) significantly gain the Raman signal, compared to the Raman measurement from pure bulk liquid water (yellow). When comparing the two 20 cm-long capillaries, more Raman signals can be detected from the aerogel-lined capillary.

The Raman signal detected from an aerogel-lined capillary increases with the increasing length of the aerogel-lined capillary. Furthermore, no disturbing interference signals from the lining itself are observed when using the aerogel-lined capillaries for Raman signal detection. 

The Raman signal gain

(1) $$G=\frac{I_{capillary}}{I_{0}}$$

was quantified as the ratio of the Raman signal intensities acquired when utilising the aerogel-lined or the silver-lined capillary *I_capillary_* (Figure 2b), and when utilising no capillary at all, *I*_0_ (Figure 2a). The Raman signal intensities *I* are the integrals of the Raman spectrum from 2900 to 3700 cm^−1^. This Raman band is due to the symmetric stretch vibration of water molecules in the liquid state of aggregation.

**Figure 5:**
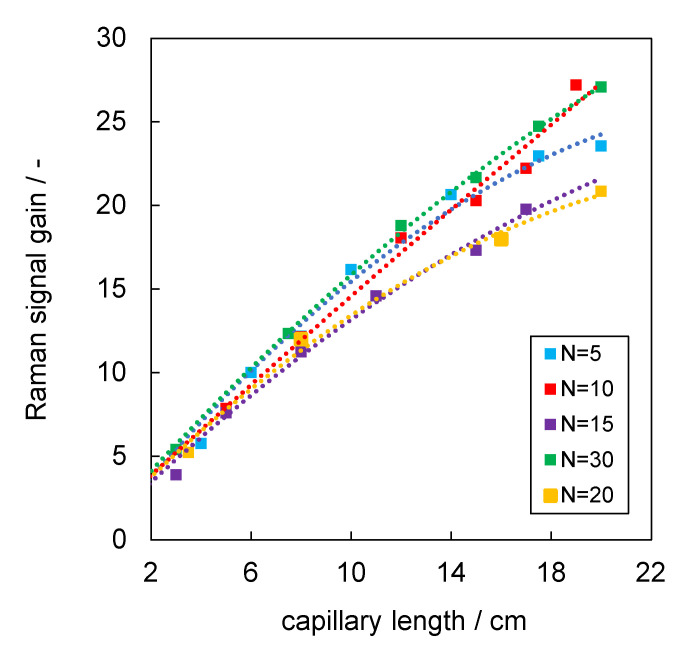
Raman signal gain G as a function of the length of the aerogel-lined capillary with an internal diameter of ∅ = 700 µm for coating repetitions between N = 5 and N = 30 (The dotted polynomial curves are to show the trend).

Figure 5 shows the Raman signal gain, computed by Equation (1), as a function of the length of the aerogel-lined capillaries for lining repetitions between N = 5 and N = 30. The largest gain was found for the longest tested capillaries. The number of coating repetitions does not influence the gain, as long as N ≥ 5. The trend in the Raman signal gain of the aerogel-lined capillaries promises that the gain can further be increased by lining longer capillaries.

Bending the aerogel-lined capillaries with radii exceeding 30 cm does not affect the Raman signal gain. We therefore conclude that the aerogel lining resists the mechanical stress caused by such bending.

### 3.3. Correlation of Raman Signal Gain and Internal Diameter of Aerogel-Lined Capillaries

As large internal diameters of aerogel-lined capillaries reduce the clogging risk, especially in industrial applications, we studied the influence of the internal diameter on the Raman signal gain.

**Figure 6:**
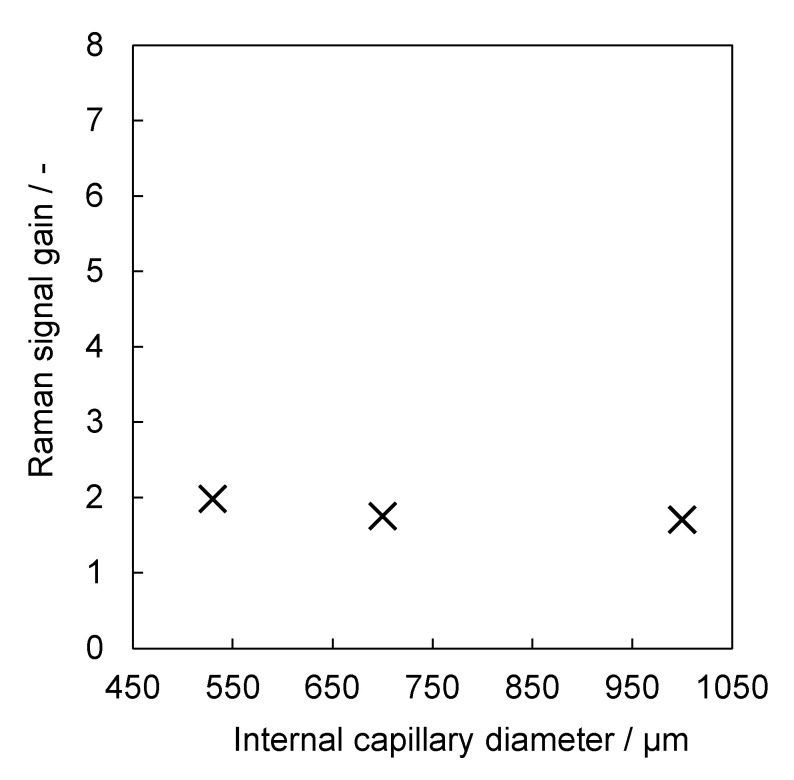
Raman signal gain G of three aerogel−lined capillaries of internal diameters of 530, 700 and 1000 µm.

It is recalled that the magnification from lens L2 to lens L3 is M_L2,L3_ = −1 and that the light-accepting area of the round end of the round-to-linear detection fibre bundle has a diameter of ∼355 µm. This implies that aerogel-lined capillaries with internal diameters exceeding 355 µm, rather than an entire liquid-core cross section, can be imaged onto the round-end interface of the detection fibre bundle. In order to assure the same detection efficiency for the aerogel-lined capillaries with internal diameters of 530 µm, 700 µm and 1000 µm, we replaced the round-to-linear detection fibre bundle with a single large-core-diameter detection fibre, whose core diameter of 1000 µm exceeds the internal diameter of the largest aerogel-lined capillary. Each aerogel-lined capillary was 20 cm long and coated N = 7 times. As can be seen from Figure 6, the Raman signal gain is not a function of the internal diameter of the aerogel-lined capillary if the entire cross section of the liquid core is imaged onto the light-accepting area of the detection fibre. However, the Raman signal gains are smaller than the ones reported in the reference. With an increasing diameter in the light-accepting area of the detection fibre, the depth of view of the Raman probe increases, as does the probed volume. Consequently, *I*_0_ in the denominator of Equation (1) increases with the increasing light-accepting area of the detection fibre, which explains why the Raman signal gains are larger when the glass fibre bundle is utilised instead of the single large-core detection fibre.

### 3.4. Raman Signal Gain Stability of Aerogel-Lined and Silver-Lined Capillaries in an Aqueous Mixture of Glucose, Ethanol and Acetic Acid

**Figure 7:**
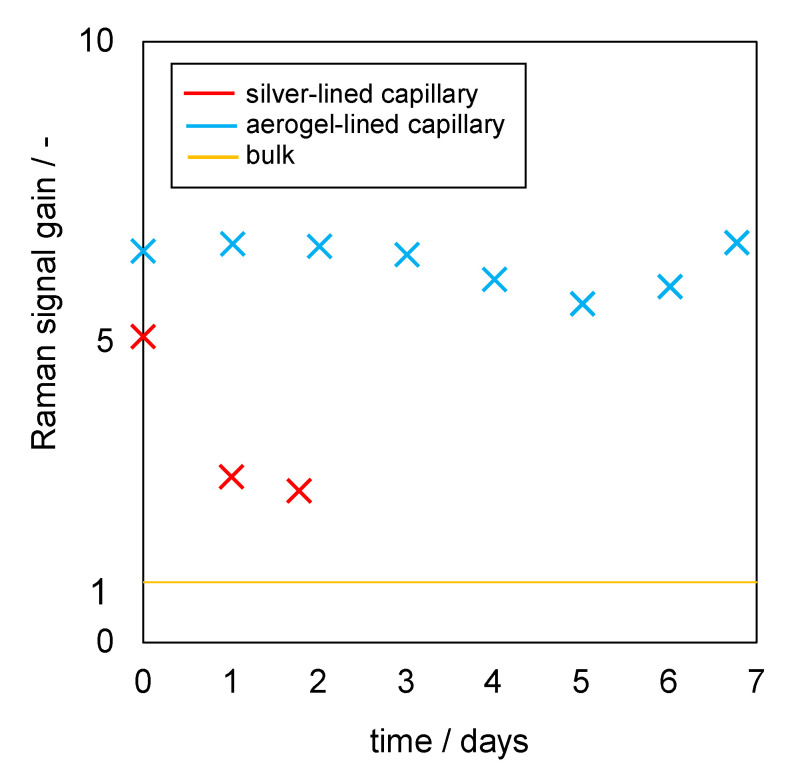
Temporal evolution of the Raman signal gain of an aerogel-lined capillary and a silver-lined capillary when exposed to a mixture of ethanol, glucose and acetic acid at pH = 4.

The Raman signal gain stability of the aerogel-lined capillaries and the silver-lined capillary was tested by exposing them for a period of seven days to a liquid mixture that mimics the stream from the production of acetic acid from biological material. In detail, the mixture was composed of ethanol (2.9 wt%), acetic acid (3.7 wt%) and glucose (4.7 wt%), and pH = 4. The aerogel-lined capillaries had an internal diameter of 700 µm, were 20 cm long and were lined N = 5 times. The silver-lined capillary had the same length and same internal diameter as the aerogel-lined capillary. The Raman signal was transferred from the Raman probe to the spectrometer via the round-to-linear fibre bundle. Figure 7 shows the relative Raman signal gain for both capillaries as a function of time. As the reproduction of aerogel linings with consistently strong TIR properties is not yet possible, the Raman signal gains reported in Figure 5 differ from the Raman signal gains reported in Figure 7 for capillaries, which are also 20 cm long.

The results show that the relative Raman signal gain from the mixture utilising the silver-lined capillary strongly decreases during the first 24 h. Thus, the long-term measurement using the silver-lined capillary was stopped after 48 h. In contrast, the Raman signal detected from the same mixture utilising the aerogel-lined capillary of the same length and same internal diameter shows no downward trend during a period of seven days. The slight variation in the Raman signal from the aerogel-lined capillary is due to light-scattering gas bubbles adhering at times to the capillary end facing the Raman probe. The crucial difference between the aerogel lining and the silver lining is the hydrophobicity of the aerogel lining, which presumably avoids direct contact with the aqueous solution, and thus protects the aerogel lining from scaling, fouling or other degradation mechanisms.

## 4. Discussion

We presented a method for gaining a Raman signal from aqueous mixtures using aerogel-lined capillaries. The aerogel linings were created by spin-coating a hydrophobised gel–particle dispersion onto fused silica capillaries of various lengths and various diameters. In pure water, 20 cm-long aerogel-lined capillaries with inner diameters of 700 µm showed that TIR properties gained a Raman signal by a factor of 27, compared to a measurement from bulk. No disturbing interference signals from the lining itself were observed. The aerogel-lined capillaries can be manufactured with large internal diameters, which make them less prone to clogging. In fact, our experiments show that the Raman signal gain does not depend on the internal diameter of the aerogel-lined capillaries in the investigated range from ∅ = 530 to 1000 µm. Moreover, it is also rather straightforward to align the laser beam into the aerogel-lined capillary due to its rather large diameter. 

We demonstrate that the Raman signal gain of aerogel-lined capillaries is durable in an aqueous mixture of sugar, ethanol and acetic acid over a period of at least 7 days. We presume that this behaviour is due to the hydrophobicity of the aerogel lining. Thus, a future task will be to investigate the role of hydrophobicity in this context. 

Although it could be demonstrated that the lining of longer fused silica capillaries promises an even higher Raman signal gain, the length of the capillaries is actually limited by the process of spin-coating. Thus, a future task will be to study alternative coating processes for aerogel-lining longer capillaries. Additional tasks will be to investigate the effect of the properties of the aerogel lining (particle size, layer thickness, refractive index, porosity, hydrophobicity, surface roughness) on its TIR properties to realise aerogel-based optofluidic waveguides with even higher Raman signal gains than 27 and to reproducibly manufacture them. 

Furthermore, we considered composite capillaries, which are a stainless-steel capillary internally lined with borosilicate glass. The internal glass lining was lined with aerogel, thus producing a three-layer composite capillary steel/borosilicate glass/silica aerogel. These composite aerogel-lined capillaries feature a mechanically robust steel body, and thus can be integrated into chemical process plants using common fittings. 

## 5. Patents

The spectroscopic analysis of liquid mixtures with a sensor that employs aerogel-lined channels as liquid core waveguides for the extension of the probed volume is covered by the patent DE 10 2019 131 698 B4.

## Data Availability

Data underlying the results presented in this paper are not publicly available at this time but may be obtained from the authors upon reasonable request.
